# Use of clomiphene citrate protocol for controlled ovarian stimulation impairs endometrial maturity

**DOI:** 10.5935/1518-0557.20200056

**Published:** 2021

**Authors:** Ivan Sereno Montenegro, Cristiana Palma Kuhl, Raquel de Almeida Schneider, Suzana de Azevedo Zachia, Isabel Cirne Lima de Oliveira Durli, Paula Barros Terraciano, Raquel Camara Rivero, Eduardo P Passos

**Affiliations:** 1 Gynecology and Obstetrics Unit, Hospital de Clínicas de Porto Alegre, Porto Alegre, Brazil; 2 Gynecology and Obstetrics Department, Universidade Federal do Rio Grande do Sul, Porto Alegre, Brazil; 3 Embryology and Cellular Differentiation Lab, Hospital de Clínicas de Porto Alegre, Porto Alegre, Brazil; 4 Pathology Unit, Hospital de Clínicas de Porto Alegre, Porto Alegre, Brazil; 5 Postgraduate Program in Health Sciences: Gynecology and Obstetrics of Universidade Federal do Rio Grande do Sul

**Keywords:** endometrial receptivity, endometrial maturity, Noyes criteria, assisted reproduction techniques, IVF

## Abstract

**Introduction:**

Despite recent advances in assisted reproduction techniques and recent knowledge regarding embryo and endometrium quality, implantation and birth rates remain low. The objective of this study was to investigate whether clomiphene citrate alters endometrial maturation in infertile patients.

**Methods:**

In a prospective self-matched cohort study, we assessed the ovulation of women in spontaneous and stimulated cycles (with clomiphene citrate). We determined the ovulation day by ultrasound scanning. In both cycles, we took four blood samples (BS1 - at early proliferative phase, BS2 - at mid proliferative phase, BS3 - after ovulation and BS4 - at mid luteal phase) to determine the serum concentrations of FSH, LH, estradiol and progesterone. We retrieved an endometrial biopsy five days after ovulation, followed by blinded analysis and classification according to Noyes criteria, in both cycles.

**Results:**

Twenty-two participants completed the study. There were significant differences in FSH BS3 (*p*=0.001), in LH BS3 and BS4 (*p*<0.001 and *p*=0.049, respectively), in estradiol BS2, BS3 and BS4 (*p*<0.001, *p*=0.024 and *p*<0.001, respectively) and in progesterone BS3 and BS4 (*p*=0.028 and *p*<0.001, respectively). Considering Noyes criteria, there was a one-day delay when comparing the stimulated cycle with the spontaneous cycle (*p*=0.004), and a two-day delay when comparing the stimulated cycle with the biopsy day.

**Conclusion:**

This study indicates that ovarian stimulation with clomiphene citrate delays the endometrial maturity, and could possibly impair the implantation process due to asynchrony.

## INTRODUCTION

Despite recent advances in assisted reproduction techniques (ART), implantation rates remain low after controlled ovarian stimulation (COS) for in vitro fertilization (IVF), and intracytoplasmic sperm injection (ICSI) treatments (van der Gaast *et al*., 2008). Embryonic implantation failure remains the major limitation of ART success (Evans *et al*., 2018; van der Gaast *et al*., 2008), and although embryo quality is the main determinant of implantation success, maturity and endometrial receptivity are important factors to consider. Embryo implantation followed by gestation depends on a viable and high quality embryo, a receptive endometrium and having a molecular "dialogue" between them (Dieamant *et al*., 2019). However, what precisely constitutes a receptive endometrium remains uncertain (Bassil *et al*., 2018; Lessey & Young, 2019; Li & Jin, 2013; Paulson, 2019). The implantation process in humans is complex and depends on multiple and successive interactions between the embryo and the endometrium, and it only succeeds when it occurs at a specific time, during the secretory phase of the menstrual cycle, called "implantation window" (Bassil *et al*., 2018; Enciso *et al.*, 2018; Lessey & Young, 2019; Li & Jin, 2013).

Recent research in this area has focused on finding a viable endometrial receptivity marker that may help to identify the best moment to perform an embryo transfer (Edgell *et al*., 2013; Evans *et al*., 2018; Kliman & Frankfurter, 2019; Miravet-Valenciano *et al*., 2015; Siristatidis *et al*., 2018). Histologically, endometrial maturity can be classified using the Noyes criteria (Kliman & Frankfurter, 2019; Noyes *et al*., 1950; Paulson, 2019), which characterizes the endometrium into its phases (Complementary table).

Clomiphene citrate (CC), commonly used in COS, is a selective estrogen receptor modulator (SERM) that has been the first-line treatment for patients with anovulation or oligomenorrhea for more than 40 years with estrogenic and antiestrogenic effects (Lindheim *et al*., 2018). It binds to estrogen receptors (ER), leading to the misinterpretation of the estradiol feedback mechanism at a hypothalamic level. The administration of this drug results in increased pituitary gonadotropin release and enhanced follicular development and ovarian response (Lindheim *et al*., 2018; Palomino *et al*., 2005).

An important CC issue is its associated low pregnancy rates, notwithstanding high ovulation rates. This phenomenon is partially explained by the antiestrogenic effect on the cervical mucus and the endometrium, causing abnormal endometrial maturation (Jie *et al*., 2018; Reed *et al*., 2018). It is therefore, tempting to hypothesize that long-lasting ER occupancy by CC might alter the endometrial cell function, thus affecting the implantation window (Palomino *et al*., 2005).

To investigate possible changes caused by CC in the pattern of endometrial receptivity, we obtained endometrial samples during the midsecretory phase from ovulatory women in a spontaneous cycle and in a CC stimulated cycle.

## MATERIALS AND METHODS

### Ethical approval

The study was developed in accordance with international laws on procedures for dealing with human tissue (including the Declaration of Helsinki for Medical Research involving Human Subjects) and STROBE's publication guidelines (Vandenbroucke *et al*., 2014). It was submitted and approved by the ethics committee of the Porto Alegre University Hospital (HCPA) and all participants signed an informed consent.

### Study population and participants

In a self-matched prospective cohort, we recruited ovulatory women from Rio Grande do Sul - Brazil, with IVF indication, between the ages of 18 and 35 years, body mass index between 18 and 30 kg/m^2^, and with tubal obstruction or male infertility. We excluded those women with anovulation, endometriosis and uterine abnormalities.

### Methodology description

**Figure 1:**
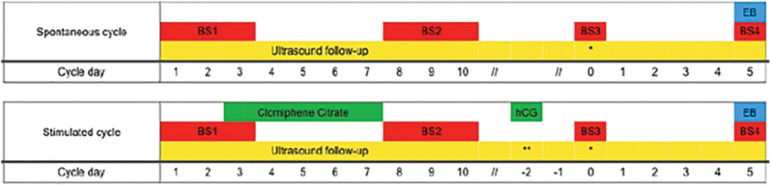
Schematic representation of the assessments in spontaneous and stimulated cycles. Following a spontaneous cycle, all 22 participants underwent a stimulated cycle using 100 mg/day of clomiphene citrate between days 3 and 7 of the cycle. When the leading follicle had a diameter of 17 mm (represented by **), they received 10.000 UI of human chorionic gonadotrophin (hCG) for final oocyte maturation. Endometrial biopsy (EB) was performed 5 days after ovulation confirmation (represented by *) by ultrasonography. In both cycles blood samples (BS) were taken at the early proliferative phase (days 1 -3), in the midproliferative phase (days 8-10), when confirmed ovulation, and five days after ovulation.

We followed the participants throughout two menstrual cycles. The first one, spontaneous, and the second, stimulated using a daily dose of 100 mg of clomiphene citrate (Clomid^®^, Sanofi, Brazil), for 5 days, initiated on the third day of the cycle. We followed the follicular growth using transvaginal ultrasound, from the second day of the cycle, until sonographic determination of ovulation. During the stimulated cycle, after identifying a follicle with 17 mm of diameter, the participant received 10.000 UI of chorionic gonadotrophin (Choriomon M^®^, Meizler UCB Biopharma S.A., Brussels, Belgic), to promote final maturation and ovulation (Figure 1).

In both cycles, collected blood samples from all participants to analyze the serum FSH, LH, estradiol and progesterone levels: first blood sample (BS1), at initial proliferative phase (days 1-3 of the cycle); second blood sample (BS2), at the mid proliferative phase (days 8-10 of the cycle); third blood sample (BS3), after determination of ovulation by ultrasound; fourth blood sample (BS4), five days after determining ovulation by ultrasound. All hormone assays were performed using electrochemiluminescent immunoassay in the Roche Cobas e602analyzer, according to correspondent kits for FSH, LH, estradiol and progesterone (FSH Elecsys and cobas e analyzers, Roche Diagnostics, USA; LH Elecsys and cobas e analyzers, Roche Diagnostics, USA; Estradiol II Elecsys and cobas e analyzers, Roche Diagnostics, USA; ProgesteroneIII Elecsys and cobas e analyzers, Roche Diagnostics, USA).

We submitted the participants to an endometrial biopsy five days after establishing the ovulation, in both cycles, with a Pipelle curette (Pipelle^®^ de Cornier for endometrial biopsy, Laboratoire C.C.D., France). We fixed the biopsy samples in 10% buffered neutral formalin, and sent them to HCPA´s pathology lab. The specimens were dehydrated with increasing concentration of ethanol before being embedded in paraffin. A portion of endometrial tissue was routinely cut, assembled and stained with hematoxylin for histological dating following the Noyes criteria (Complementary table) (Noyes *et al*., 1950). The same blinded examiner (RCR), an expert in gynecological pathology at HCPA, evaluated all slides and the result was expressed in whole numbers, using as a reference, a standard menstrual cycle of 28 days of duration and ovulation occurring on the 14^th^ day of the cycle.

### Statistical analyses

FSH, LH, estradiol and progesterone samples were compared using a Generalized Estimating Equation (GEE) model concerning values between collections, cycles and interaction (collection x cycle). We presented the data as mean ± standard error. For the calculation model, we established the following parameters: unstructured working correlation matrix, robust estimator covariance matrix, and normal distributions with identity function. When significant, we used the Bonferroni post-hoc test to ascertain which blood samples were different. We analyzed the histopathological results of Noyes criteria using the Wilcoxon test for related samples, which compares the distribution of the histopathological data of the endometrial matched samples. We expressed this data using median values (25-75). For all tests, a *p*<0.05 was significant.

## RESULTS

### Demographic data

**Figure 2:**
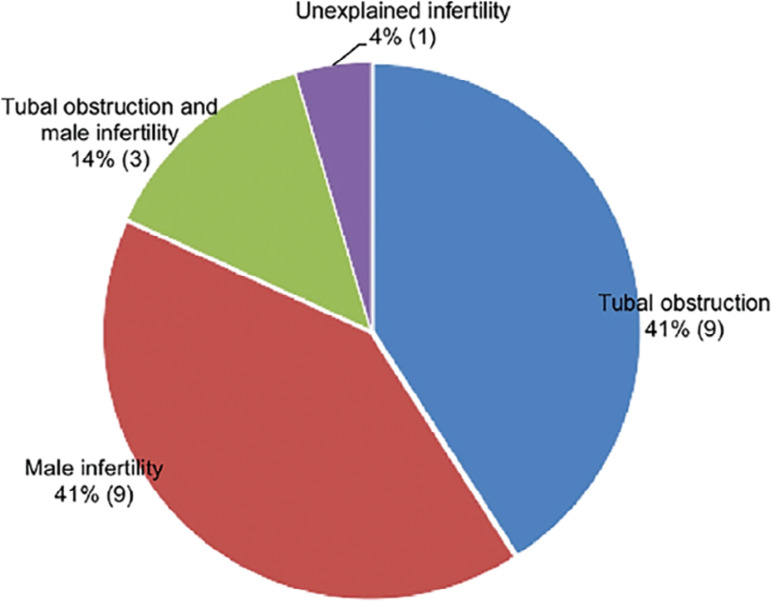
Participants’ causes of infertility.

We recruited thirty-six patients and, of these, 22 completed the two assessment cycles and were included for analysis of the data collected, description (presented in the Table 1 and Figure 2) and statistical evaluation.

**Table 1:** Participant demographic data (n=22)

Data	Mean ± SD
Age (years)	31.5±3.5
Weight (Kg)	64.5±6.93
Height (m)	1.62±0.06
Body mass index (Kg/m^2^)	24.53±3.02

SD - Standard derivation

### Hormonal analysis

Table 2 shows the mean serum levels of the hormones FSH, LH, estradiol and progesterone. When comparing mean values between the corresponding samples for each cycle, there were differences in the FSH dosage comparisons from BS3 (*p*=0.001); in LH, from BS3 (*p*<0.001) and BS4 (*p*=0.049); in estradiol from BS2 (*p*<0.001), BS3 (*p*=0.024) and BS4 (*p*<0.001); and in progesterone, from BS3 (*p*=0.028) and BS4 (*p*<0.001).

**Table 2:** Comparison of hormonal dosages ^†^

Hormone		Spontaneous Cycle	Stimulated Cycle	Total	*p* Cycle	*p* Sample	*p* Interaction
		Mean (SE)	Mean (SE)	Mean (SE)			
FSH	Sample 1	7.62 (0.51)	7.02 (0.41)	7.32 (0.41)	0.023	<0.001	**0.002**
	Sample 2	6.21 (0.56)	6.69 (0.74)	6.45 (0.62)			
	Sample 3	**6.83a (0.62)**	**9.75b (0.82)**	8.29 (0.58)			
	Sample 4	3.64 (0.22)	3.90 (0.36)	3.77 (0.21)			
	Total	6.08 (0.30)	6.84 (0.42)				
LH	Sample 1	6.26 (0.66)	6.27 (0.59)	6.26 (0.60)	<0.001	<0.001	**0.002**
	Sample 2	8.85 (0.96)	9.97 (0.84)	9.41 (0.80)			
	Sample 3	**12.50a (1.99)**	**22.26b (2.50)**	17.38 (1.89)			
	Sample 4	**6.68a (0.65)**	**8.33b (1.06)**	7.50 (0.77)			
	Total	8.57 (0.79)	11.71 (0.92)				
Estradiol	Sample 1	40.10 (5.31)	38.90 (2.37)	39.50 (3.39)	<0.001	<0.001	**<0.001**
	Sample 2	**127.08a (14.17)**	**480.87b (54.13)**	303.97 (29.56)			
	Sample 3	**111.43a (13.05)**	**163.97b (16.31)**	137.70 (9.15)			
	Sample 4	**155.03a (14.76)**	**481.22b (41.30)**	318.13 (21.81)			
	Total	108.41 (6.65)	291.24 (23.25)				
Progesterone	Sample 1	0.65 (0.19)	0.49 (0.12)	0.57 (0.12)	0.002	<0.001	**<0.001**
	Sample 2	0.44 (0.07)	0.35 (0.06)	0.39 (0.05)			
	Sample 3	**4.87a (0.79)**	**2.99b (0.43)**	3.93 (0.47)			
	Sample 4	**13.37a (0.78)**	**25.86b (2.75)**	19.62 (1.42)			
	Total	4.83 (0.32)	7.42 (0.76)				

SE -Standard Error Distinct letters represent statistically different means

† Generalized Estimating Equation Model (GEE)

### Endometrial analysis

**Figure 3:**
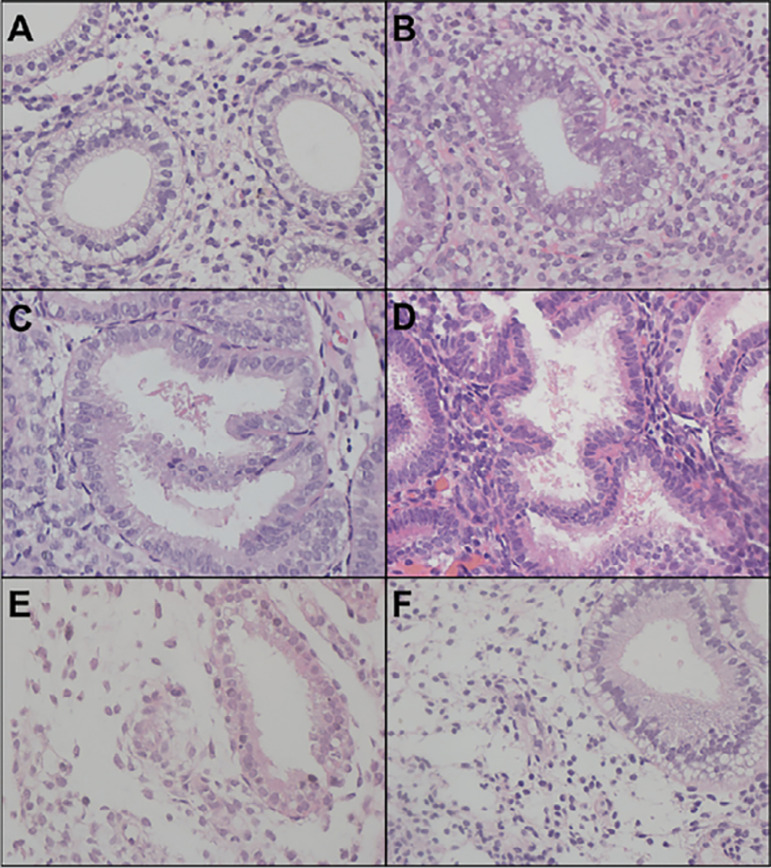
Endometrial cycle dating based on Noyes et al. (1950) criteria (200x): (A) Day 17: “piano key” appearance of glandular epithelium with vacuoles at nuclei level; (B) Day 18: luminal vacuoles with smaller size and the nuclei approaching the base of the glandular cell; (C) Day 19: presence of intraluminal secretion with few vacuoles on cellular cytoplasm, absence of mitosis and pseudo stratification; (D) Day 20: the peak of intraluminal secretion with stromal edema onset, presence of rare vacuoles and round nuclei located at the base of the glandular cell.; (E) Day 23: presence of prominent spiral arterioles with thickened walls, coiling and endothelial proliferation; (F) stromal-glandular dissociation - gland on day 17 with decidualized stroma.

**Figure 4:**
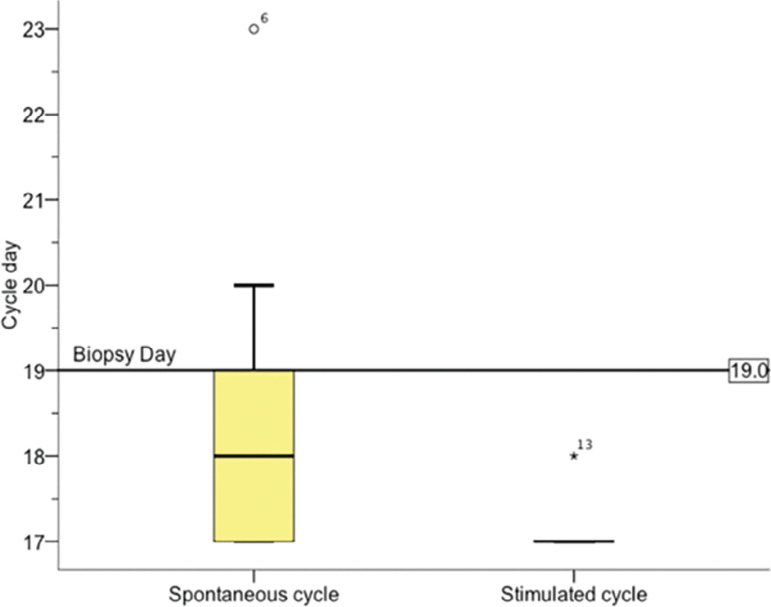
Median comparison of Noyes criteria results.

We evaluated eighteen endometrial matched samples by Noyes criteria tissue analysis (Figure 3). Four pairs of samples were withdrawn due to results that showed, at least in one slide, a stromal-glandular dissociation (2 slides), a basal endometrium (1 slide) or could not be evaluated (1 slide). The comparison between the median results of the endometrial biopsy from both cycles expressed a difference (*p*<0.004) of one day (Figure 4).

## DISCUSSION

This study found a one-day delay in the CC stimulated cycle, when analyzing the endometrium following the Noyes criteria. This data supports the idea of asynchrony between the endometrium in the spontaneous and stimulated cycles. Concerning the day of blastocyst transfer, in other words, 5 days after ovulation, there was a 2-day delay - which could explain even lower implantation rates when performing a transfer on this day in cycles using CC. It was the first to study that analyzed endometrium matched samples in spontaneous and stimulated cycles from the same infertile women.

Some investigators report on the lack of correlation between the cycle day and endometrial dating by Noyes criteria. Murray *et al.* (2004) concluded that histologic endometrial dating does not have the accuracy or the precision necessary to provide a valid method for the diagnosis of luteal phase deficiency and, Coutifaris *et al*. (2004) stated that the endometrial biopsy followed by histological dating provides no useful clinical information as a screening test for infertility. Garrido-Gómez *et al*. (2013) said that biochemical markers are ideal as alternatives to classic Noyes criteria and suggest that the definition of a genomic signature of human endometrial receptivity can be used as a strategy to overcome subjectivity problems caused by the inter and intracycle variations in Noyes endometrial receptivity dating. In 2018, Bassil *et al*. found no agreement between the endometrial receptivity array (ERA) and the Noyes histological criteria (Bassil *et al*., 2018). Despite these relevant findings, they used different methods to reach this conclusion (none of them using the same participant in a spontaneous and in a stimulated cycle) and there are no other validated markers or clinically useful endometrial evaluation methods.

It should be noted that our participants are diagnosed with infertility and with indication of IVF; however, the ones with anovulation, uterine abnormalities and endometriosis were carefully excluded to minimize possible selection biases, as it is considered that these pathologies may modify the endometrial decidualization process. Yet, we biopsied all the participants in the spontaneous cycle and in a CC-stimulated cycle, minimizing other types of selection biases.

The significant increases in FSH, LH, estradiol and progesterone dosage confirm that participants correctly used the medication provided. As known, CC works by competitive binding to estrogen receptors in the hypothalamus and pituitary, reducing estrogen signaling via receptors and interfering with the feedback mechanism of endogenous estrogen, resulting in an increase in FSH and LH secretions to stimulate ovarian follicular production, and a consequent increase in estradiol and progesterone (Gadalla *et al*., 2018). One of our hypotheses is that the supraphysiological increase in the estradiol, progesterone, FSH and LH levels in stimulated cycles with clomiphene citrate, may be one of the factors responsible for the asynchrony of endometrial histology.

Considering the 22 matched samples of endometrial biopsies, we excluded four pairs from the statistical calculation, two histological results demonstrated a stromal-glandular dissociation and another one a basal endometrium. These findings confirm that CC use for COS causes asynchrony and delayed endometrial development, although it was not used for analysis.

Bonhoff *et al*. (1996) described the same asynchrony effect in patients undergoing COS with CC for artificial insemination, comparing them with fertile controls. Gonzalez *et al*. (2001) also reported an endometrial asynchrony and integrins alteration in CC. stimulated cycles and intrauterine device users, when compared to fertile controls.

Looking at these results, we hypothesize that the antiestrogenic effects of CC on the endometrium impairs endometrial proliferation in the proliferative phase of the cycle, which reflects a delayed endometrial maturation in the luteal phase. This finding is confirmed by several studies which concluded that ovulation induction with CC might result in lower endometrial thickness (Gadalla *et al*., 2018; Reed *et al*., 2018) and, despite the high rate of ovulation, pregnancy rates are low when using CC (Jie *et al*., 2018).

There are few limitations to our study. We did not compare these findings to implantation, pregnancy and birth rates, as we consider these outcomes the best predictors for any developed endometrial receptivity test. Despite the small sample of this study, our results show a significant difference in endometrial maturity comparing the spontaneous and stimulated cycle with CC. Regardless of the Noyes criteria limits, there are no other validated test to assess endometrial maturity, so that we use the same test, performed by the same blinded observer, in samples of the same participant in order to minimize this bias.

In conclusion, our study found statistically significant changes in the endometrial compartment in patients who used clomiphene citrate for ovarian stimulation, represented by an asynchrony demonstrated by a one-day delay in histological endometrial maturity when comparing spontaneous and stimulated cycles; and a two-days delay when compared the stimulated cycle with the probable day of blastocyst transfer. The freeze-all strategy is a possible approach to be adopted in IVF cycles, to avoid this asynchrony with subsequent transfer in a spontaneous cycle or associated with endometrial preparation. In order to confirm this finding, we need larger prospective studies.

## Complementary table

**Complementary table:** Noyes criteria

Endometrium phase or days	Histopathological characteristics of the endometrium
*Proliferative phase*	Length varies from 10 - 20 days, "ideal" is 14 days During this phase, glands become more tortuous due to epithelial proliferation, in response to estrogen production and estrogen receptors on epithelium
Early proliferative (days 4 - 7)	Thin surface epithelium, **straight** short glands, compact stroma, minimal mitotic activity and large nuclei
Mid proliferative (days 8 - 10)	Columnar surface epithelium; longer **curving glands**, variable **stromal edema** and numerous mitotic figures
Late proliferative (days 11 - 14)	Undulant surface epithelium, tortuous glands with prominent mitotic activity and pseudostratification; dense stroma, subnuclear vacuoles in less than 50% of glands
*Ovulation*	presence of subnuclear vacuoles in 50% of glands is evidence of ovulation; must biopsy the functional layer, not the basal layer; to rule out anovulatory cycles, should biopsy 2 days before menstruation
*Secretory / luteal phase*	Traditionally assumed to be 14 days, but may vary Progesterone secretion inhibits endometrial proliferative activity and induces secretory activity Note: secretory material in glands is NOT specific for secretory epithelium; seen also in disordered proliferative and hyperplastic endometrium and carcinoma Clinically, secretory endometrium is lush and polypoid with no necrosis; may be hemorrhagic if close to day 28
Day 15	No changes from late proliferative; also known as interval endometrium; presence of scattered nuclear vacuoles is not specific for ovulation (must be 50% or more)
Day 16	"Piano key" appearance; subnuclear vacuoles
Day 17	"Piano key" appearance; vacuoles at level of nuclei
Day 18	Luminal vacuoles, smaller size and nuclei approach base of cell
Day 19	Intraluminal secretion begins
Days 20 - 21	Maximal secretion
Day 22	Maximal stromal edema in luteal phase; best time for implantation
Day 23	Prominent spiral arterioles (thickened walls, coiling and endothelial proliferation)
Day 24	Perivascular predecidualization (stromal cell hypertrophy with accumulation of cytoplasmic eosinophilia); serrated / tortuous glands
Day 25	Predecidualization below surface endometrium
Day 26	Confluence of predecidual tissue; stromal granulocytes (probably lymphocytes) appear
Day 27	Prominent stromal granulocytes; focal necrosis and hemorrhage
Day 28	Shedding, also called glandular and stromal breakdown; prominent necrosis and hemorrhage; predecidual stroma and glandular exhaustion; nuclear dust at base of glandular epithelium; condensed stroma with overlying papillary-syncytial change; intravascular fibrin thrombi; stromal granulocytes

Ref: Noyes *et al*., 1950
